# Supplementary material for: Does women empowerment associate with reduced risks of intimate partner violence in India? evidence from National Family Health Survey-5
Source: PLoS One. 2023 Nov 28;18(11):e0293448. doi: 10.1371/journal.pone.0293448 (PMC10684075; doi:10.1371/journal.pone.0293448)
Supplement: S1 Table — (DOCX) [file pone.0293448.s001.docx]

**Supporting Information**

**S1 Table.
Questions asked to married women on their experience of different forms of IPV**

| **Type of violence** | **Question asked to married women (18-49 years)** |
| --- | --- |
| **Physical violence** | Did your (last) husband –  – Push you, shake you, or throw something at you? |
|  | – twist your arm or pull your hair? |
|  | – slap you? |
|  | – punch you with his fist or with something that could hurt you? |
|  | – kick you, drag you or beat you up? |
|  | – try to choke you or burn you on purpose? |
|  | – threaten or attack you with a knife, gun, or any other weapon? |
| **Sexual violence** | Did your (last) husband –  – physically force you to have sexual intercourse with him even when you did not want to? |
|  | – physically force you to perform any other sexual acts you did not want to? |
|  | – force you with threats or in any other way to perform sexual acts you did not want to? |
| **Emotional violence** | Did your (last) husband –  – say or do something to humiliate you in front of others? |
|  | – threaten to hurt or harm you or someone close to you? |
|  | – insult you or make you feel bad about yourself? |
