# Supplementary material for: Does women empowerment associate with reduced risks of intimate partner violence in India? evidence from National Family Health Survey-5
Source: PLoS One. 2023 Nov 28;18(11):e0293448. doi: 10.1371/journal.pone.0293448 (PMC10684075; doi:10.1371/journal.pone.0293448)
Supplement: S2 Table — (DOCX) [file pone.0293448.s002.docx]

**Supporting Information**

**S2 Table.
Composite IPV scores (ranked from highest to lowest) versus the corresponding composite women empowerment score**

| **State** | **Intimate Partner Violence** (from highest to lowest) | **Women  Empowerment** |
| --- | --- | --- |
| Karnataka | 3.0 | 3.73 |
| Bihar | 2.31 | 1.77 |
| West Bengal | 2.04 | 2.11 |
| Telangana | 1.95 | 4.21 |
| Uttar Pradesh | 1.87 | 2.05 |
| Assam | 1.84 | 2.39 |
| Ladakh | 1.82 | 3.65 |
| Manipur | 1.74 | 4.03 |
| Jharkhand | 1.73 | 2.78 |
| Madhya Pradesh | 1.72 | 1.61 |
| NCT of Delhi | 1.62 | 3.07 |
| Arunachal Pradesh | 1.60 | 3.72 |
| Maharashtra | 1.56 | 2.61 |
| Andhra Pradesh | 1.55 | 3.15 |
| Odisha | 1.51 | 3.01 |
| Tamil Nadu | 1.46 | 4.75 |
| Tripura | 1.43 | 1.93 |
| Meghalaya | 1.38 | 3.28 |
| Rajasthan | 1.36 | 2.17 |
| Haryana | 1.12 | 2.45 |
| Chhattisgarh | 1.08 | 2.87 |
| Sikkim | 1.05 | 3.87 |
| Puducherry | 1.03 | 4.95 |
| Uttarakhand | 0.91 | 2.83 |
| DDN and Daman & Diu | 0.89 | 3.89 |
| Gujarat | 0.83 | 2.28 |
| Jammu & Kashmir | 0.80 | 2.81 |
| Goa | 0.76 | 4.21 |
| Punjab | 0.73 | 3.67 |
| Himachal Pradesh | 0.56 | 3.38 |
| Mizoram | 0.55 | 3.80 |
| A & N Islands | 0.54 | 3.83 |
| Kerala | 0.54 | 3.63 |
| Chandigarh | 0.52 | 3.60 |
| Nagaland | 0.43 | 2.93 |
| Lakshadweep | 0 | 2.85 |
