# Supplementary material for: Does women empowerment associate with reduced risks of intimate partner violence in India? evidence from National Family Health Survey-5
Source: PLoS One. 2023 Nov 28;18(11):e0293448. doi: 10.1371/journal.pone.0293448 (PMC10684075; doi:10.1371/journal.pone.0293448)
Supplement: Attachment — Submitted filename: Response to Reviewers-17July.docx [file pone.0293448.s003.docx]

**Response to Reviewer Comments**

**PONE-D-23-02776**

**Do empowered women have lower risks of intimate partner violence in India? Evidence from National Family Health Survey-5**

------------------------------------------------------------------------------------------------------------

Dear editor,

We are thankful to your office, and the two reviewers for supporting us in improving the quality of the manuscript. We have put all comments in a tabular format, and each comment has a corresponding response. Also, all changes in the main manuscript are in track change.

| **Comment by Academic Editor** | | **Response  [highlighted in pink in manuscript]** |
| --- | --- | --- |
| 1 | Please ensure that your manuscript meets PLOS ONE's style requirements, including those for file naming. | All referencing and formatting style aligned with journal requirement. |
| 2 | Please provide additional details regarding participant consent. In the ethics statement in the Methods and online submission information, please ensure that you have specified what type you obtained (for instance, written or verbal, and if verbal, how it was documented and witnessed). If your study included minors, state whether you obtained consent from parents or guardians. If the need for consent was waived by the ethics committee, please include this information | The required ethics statement **has been added** to the Method’s ethical considerations and data quality section, including a reference:  [“Additional details on the protocol are available at <https://dhsprogram.com/Methodology/Survey-Types/DHS.cfm>.”] |
| 3 | In your Data Availability statement, you have not specified where the minimal data set underlying the results described in your manuscript can be found. PLOS defines a study's minimal data set as the underlying data used to reach the conclusions drawn in the manuscript and any additional data required to replicate the reported study findings in their entirety. All PLOS journals require that the minimal data set be made fully available.  Upon re-submitting your revised manuscript, please upload your study’s minimal underlying data set as either Supporting Information files or to a stable, public repository and include the relevant URLs, DOIs, or accession numbers within your revised cover letter. | Sorry for not being clear on this. All the Summary Fact Sheets and data used in the study is available on the DHS and NFHS website through their portal for registered users. We have **made the edits** on the DATA AVAILABILITY statement.  (<https://dhsprogram.com/data/http://rchiips.org/nfhs/factsheet_NFHS-5.shtml>) |
| 4 | We note that Figure 1 in your submission contain map images which may be copyrighted. All PLOS content is published under the Creative Commons Attribution License (CC BY 4.0), which means that the manuscript, images, and Supporting Information files will be freely available online, and any third party is permitted to access, download, copy, distribute, and use these materials in any way, even commercially, with proper attribution. For these reasons, we cannot publish previously copyrighted maps or satellite images created using proprietary data, such as Google software (Google Maps, Street View, and Earth). For more information, see our copyright guidelines: <http://journals.plos.org/plosone/s/licenses-and-copyright>.  We require you to either (1) present written permission from the copyright holder to publish these figures specifically under the CC BY 4.0 license, or (2) remove the figures from your submission | Figure-1 images have now been modified once more.  We have done visualization using **opensource technology R**, which is a programming language and environment for Statistical computing and graphics. We used ggplot2 package of R for this purpose in the R studio for Windows. We believe this is in compliance with PLOS policy. |
| 5 | Please review your reference list to ensure that it is complete and correct. If you have cited papers that have been retracted, please include the rationale for doing so in the manuscript text, or remove these references and replace them with relevant current references. Any changes to the reference list should be mentioned in the rebuttal letter that accompanies your revised manuscript.  If you need to cite a retracted article, indicate the article’s retracted status in the References list and also include a citation and full reference for the retraction notice. | We have added 3 references (**refs # 37, 38, 39**).  We have updated the Reference list as per the journal’s referencing style policy (Vancouver). |
| **Comments by Reviewer # 1** | | **Response  [highlighted in blue in manuscript]** |
| 1 | The first statement of the abstract contradicts the last statements of the first and last paragraphs of the background. | Yes indeed. Thank you. We have now **edited the starting part of the Abstract** to be synchronous with the Background. Background is also edited to be consistent. |
| 2 | The aim of the study is found in the fourth paragraph of the background but the authors have aim among others as a sub-heading in the method. | Yes, indeed we had duplicated it. We have **now removed** ‘aim’ from under Methods section. |
| 3 | Lakh is used several times. It would help readers if the English meaning of Lakh could be put in brackets immediately after using it. | We have decided to just **use the numbers**, since in millions, the numbers are less than even one million. |
| 4 | There are two sub-headings under the method that have “design”. The authors need to correct it. | Thank you for pointing that out. The redundant ‘Design’ word was **removed** from the Method section. |
| 5 | The authors listed Microsoft Office Excel-2021, R Studio, and SPSS version 22.0 as the softwares used to analyse the data. There is a need for an explanation to be given as to how each software was used. | The appropriate explanation was **added** to the Statistical Analysis section. |
| 6 | A summary of descriptive statistics of the variable could be given in the result section before the correlation matrix. This would throw more light on the manuscript. | We have **re-ordered** the Results section to address the reviewer comment.  The Result section now has the description (heatmap) first, followed by the table of Composite Score, followed by the Correlation Matrix. |
| 7 | If the authors attend to these concerns, the article will provide very relevant information for academics and the policymakers. | Thank you; we have tried our best to address all the concerns to the best of our capabilities. |
| **Comments by Reviewer # 2** | | **Response  [highlighted in yellow in manuscript]** |
| 1 Title: | I will suggest that the authors reframe the topic as: Are Women’s’ Household decision-making, Mobile Phone Ownership, and Menstrual Hygiene associated with reduced IPV? Findings from NFHS-5, India. | We have now **changed the title** to make it broader. Detailed response related to the Title is also under point # 6 below.  [Revised title is – Do empowered women have lowered risks of intimate partner violence in India? Evidence from National Family Health Survey- 5] |
| 2 Abstract | The background of the abstract simply ignored other important aspects of the work that were highlighted in the topic. Hence, the other parts such as mobile phone ownership and menstrual hygiene will need to be discussed **unless** they are simply measures of women’s household decision-making. | We confirm that for this manuscript, mobile phone ownership and menstrual hygiene were our findings, **not** part of the aim. So, we have **changed the title** to be compatible with the Aim, and not Findings.  Thus, we did **not** edit the Background (Abstract) to include mobile phone ownership or menstrual hygiene issues. We have left it at discussing women empowerment and its likely association with IPV, and that we are aiming to study the association for India. |
| 3 Abstract | The method section of the abstract should include the sample size for this study, it’ll be important to show that. | Agree with the reviewer. Details about sample size are now **added**. |
| 4 Abstract | Intimate Partner Violence should be included as a keyword. | Sure, **included** now. |
| 5 Introduction | The last set of sentences on the relationship between employment and IPV risk (Pages 3-4) can be communicated in a single sentence. | Thank you. The sentences have been **merged**, and the references **repositioned** accordingly. |
| 6 Introduction | Similar to the background section of the abstract, the introduction does not depict what the topic is communicating. The authors will need to decide whether this study is entirely focused on women’s empowerment and IPV, with mobile phone ownership and menstrual hygiene as measures of women’s empowerment or rewrite the whole introduction section to incorporate ideas on mobile phone ownership//menstrual hygiene and their links with IPV. | This is indeed a significant discrepancy in the manuscript – thank you for highlighting it.  Co-authors had a round of discussion and we realise that specifically mentioning mobile phones or menstrual hygiene in the title is ***not*** what we want to do. The paper set out to study the association with women empowerment and IPV (as stated in the aim) and found these variables as significant. Not the other way round. So, we have decided to **change the title** so that it is broader and reflects our primary question, and not the findings.  So, we have **not** edited the Introduction to highlight literature on mobile phones and menstrual hygiene but kept the focus on empowerment and its broad indicators only, in terms of literature review in this Introduction. |
| 7 Method | Excellent description of the method section especially the statistical analyses employed in this study. However, the authors should endeavor to provide some more information on the study design, especially the type of sampling and how the NSFH study respondents were selected. | Thank you for your appreciation. We have now **added** the detailed sampling and the selection of sample size. |
| 8 Results, Discussion, and Conclusion | The results were greatly presented, and the findings were discussed in light of similar findings in the literature. | Thank you. |
| 9 Results, Discussion, and Conclusion | It would be highly appropriate for the authors to offer suggestions for future studies in the context of the study. | Yes, we agree. We have **added** a paragraph on future research pathways at the end of the Discussion section. |

*We are indeed grateful to all reviewers for taking time out to review this manuscript. Some of us do anonymous reviews as well and do understand and deeply appreciate what it takes to do such a thorough review. Do accept our sincere thanks.*
