# Supplementary material for: Does women empowerment associate with reduced risks of intimate partner violence in India? evidence from National Family Health Survey-5
Source: PLoS One. 2023 Nov 28;18(11):e0293448. doi: 10.1371/journal.pone.0293448 (PMC10684075; doi:10.1371/journal.pone.0293448)
Supplement: Attachment — Submitted filename: [AdeoyePA review] Revised Manuscript without track changes PLOS ONE IPV.docx [file pone.0293448.s004.docx]

**Do empowered women have lowered risks of intimate partner violence in India? Evidence from National Family Health Survey-5**

Rakhi Ghoshal^1^, Priti Patil^2^, Anita Gadgil^3^, Priyansh Nathani^4^, Prashant Bhandarkar^2^,
Dnyaneshwar B. Kale^5^, Nobhojit Roy^6,7*^

^1^ Gender Equality Centre, CARE India, New Delhi, India

^2^ Department of Statistics, BARC Hospital, Mumbai, India.

^3^ Department of Surgery BARC Hospital, Mumbai, India.

^4^ Hinduhridaysamrat Balasaheb Thackeray Medical College and Dr Rustom Narsi Cooper Municipal General Hospital, Mumbai, India.

^5^ Indian Institute of Health Management Research, Rajasthan, India

^6^ Department of Global Public Health, Karolinska Institutet, Sweden

^7^ The George Institute for Global Health, New Delhi, India

* Corresponding author:

Email: [nobhojit.roy@ki.se](mailto:nobhojit.roy@ki.se) (NR)

**Abstract**

**Background**

Women empowerment is commonly believed to be an important factor affecting a woman’s likelihood of facing violence from her intimate partner. Even as countries invest in policies that aim to strengthen women empowerment, studies show that increased empowerment does not decrease intimate partner violence (IPV) against women. Against this paradox, the present study seeks to understand the empowerment components that associate with IPV against women in India. It studies the state-level distribution of IPV in its disaggregated forms.

**Methods**

The study analyses state-level data from the National Family Health Survey, India (2019-21). A total of 72,056 women responded to the domestic violence questionnaire. The Dimension Index (DI) was used to compute composite scores for Women Empowerment and for IPV to rank states and Union Territories. The correlation between Women Empowerment and IPV scores was determined using Spearman's rank correlation coefficient.

**Results**

The Indian state of Karnataka had the highest composite score of IPV and showed the highest disaggregated burden for physical, sexual and emotional IPV. Physical IPV was the most common form of IPV for most states. The Western states of India showed up with lowered burdens for all three types of IPV. The specific three components of empowerment, viz. household decision-making, mobile phone ownership and hygienic menstrual practices associated with reduced IPV. However, property ownership increased the risk. Emotional IPV was lower for women who participated in household decision-making, while emotional and sexual IPV increased with property ownership.

**Conclusions**

The study found no significant reduction in IPV with improvement in women empowerment. However, the study identifies the components of empowerment that associate with a likelihood of reduced IPV. The findings of this study would be able to inform future research and intervention that seek to strengthen these specific components of building women empowerment.

**Keywords**

Intimate partner violence; Domestic violence; Employment; Decision-making; Gender-based violence; Spousal violence; Male backlash; Ownership of asset

# Do empowered women have lowered risks of intimate partner violence in India? Evidence from National Family Health Survey-5

**Background**

Violence inflicted by one’s intimate partner or the spouse (Intimate Partner Violence or IPV) is a global problem with severe physiological and psychological health consequences (1). Globally, one in three women face IPV at least once in their lifetime (2). While there are several factors associated with IPV (3)one of the most examined factors is women empowerment (4). The components of women empowerment include level of education, ability to take household decisions and access to material resources (4) (5). Empowerment is commonly assumed to reduce the likelihood of IPV in a woman’s life (6).

Certain countries including India, have put in place policies and legislations that prioritise strengthening the different components of women empowerment. Legal provisions and Acts such as the Maternity Benefit Act (1961), the Equal Remuneration Act (1976), Protection of Women from Domestic Violence Act (2005), and the Prohibition of Sexual Harassment of Women at Workplace Act (2013) among others, empower women across different domains of life. If observed longitudinally, women empowerment has increased in India (7). However, studies continue to find that empowerment components such as employment increase the risk of IPV for women (3,8) (5) up to nine times (9). Data over a decade found that women with higher levels of education, and with increased economic control over household resources were more vulnerable to IPV (10). Studies that looked at women empowerment and IPV in the Indian context – particularly those using the population level cross-sectional National Family Health Survey (in its various rounds from 1992-93, 1998-2000, 2005-06 and 2015-16) (5,8–10) – found that IPV against women had not reduced with increase in women empowerment (8,11).

The present study was undertaken to evaluate the association of women empowerment with IPV against women in India. It also studies the state-level distribution of IPV (disaggregated as physical, sexual and emotional IPV). Pursuing this question is important since women empowerment is associated with improved chances of women’s education, which in turn is known to delay the age of marriage for women and lower their fertility, increasing their reproductive autonomy (12). Empowered women are also more likely to plan their pregnancies and better utilize maternity care (13). They also have more equality among spouses (14). A range of positive changes nonetheless unfold when women empowerment increases.

**Method**

**Design and setting**

This study offers a state-level analysis of the association between women empowerment and risk of IPV. It uses cross-sectional data that was collected as part of the household level survey, the National Family Health Survey-5, between 2019 and 2021. The National Family Health Survey (NFHS) is modelled on the Demographic and Health Survey (DHS), and captures a wide range of health parameters, including data on Women Empowerment and Domestic Violence. The data is captured at the state and district levels, and supports policymakers, implementors and administrators to design implementations that are informed by evidence. The 5^th^ round of the NFHS is the latest round and includes representative urban and rural household samples from all 28 states and 8 Union Territories (U.T.) across the country.

**Sample size**

In this round of the NFHS data was collected from 636,699 households which included 724,115 women and 101,839 men. The Domestic Violence questionnaire module was administered to married women in the age group 18-49^[1]^ years. To adhere to ethical guidelines, only one eligible woman per household was interviewed. Special weights were applied to account for the selection of only one woman per household and to ensure that the subsample related to domestic violence was representative at the national level. A total of 72,056 women provided responses to the domestic violence module.

**Independent variables: women empowerment**

We considered Women Empowerment as an independent variable. Empowerment was further disaggregated by the NFHS as, percentage of women (a) participating in major household decisions, (b) who worked during last year and were paid in cash, (c) who own house and/or land, (d) have an own bank account, (e) who have an own mobile phone, and (f) who use hygienic methods of protection during menses.

**Outcome variable: women facing IPV**

IPV faced by ever-married women was further disaggregated into physical, sexual and emotional IPV. Percentage of women who faced either of the three types of IPV was the dependent variable and was calculated per state/U.T. The questionnaire for the Domestic Violence schedule is available as a Supporting Information Table S1.

**Statistical analysis**

Statistical analysis was carried out using Microsoft office Excel-2019, R Studio, and SPSS version 22.0 (SPSS Inc., Chicago, IL, USA) for windows for doing statistical analysis. NFHS Data factsheet were extracted in comma separated value (CSV) file format which were further summarized for the analysis. Heat-map-based visualization is done using opensource technology R, which is a programming language and environment for Statistical computing and graphics. We used ggplot2 package of R for this purpose in the R studio for Windows. We used the Dimension Index (DI) measures developed by Iyengar and Sudarshan (15). The spatial aspects of the development are used to measure the level or stage of the state’s development. This method was preferred since it requires less assumptions compared to other methods such as Principal Component Analysis. The Dimension Index (DI) was calculated separately for each state and UT for all the independent variables.

$Dimension Index (DI)=\frac{Actual Value of the Indicator-Minimum Value}{Maximum Value-Minimum Value}$

For e. g. DI for the state of Bihar for physical IPV is calculated as:

$$DI\_Bihar=\frac{Physical Violence \% for Bihar-Minimum Physical Violence \% across all states and UTs}{Maximum-Minimum values of the Physical Violence across all states and UTs}$$

The DI value lies between 0 and 1. A greater value indicates a higher rate. For any of the component of Women Empowerment, the closer a value is to 1, the better women empowerment is for that state. When calculating for IPV, the closer a value is to 1, the bigger the burden of IPV is for that state. The DI calculated composite scores for Women Empowerment and also for IPV. The composite score is the addition of the DI of respective indicators. The states and UTs were ranked according to the score.

Spearman’s rank correlation coefficient is used to find the association between Women Empowerment and IPV composite scores. The standard convention of Dancey and Reidy (16) was used for the interpretation of Spearman coefficient values and grouped the association as either weak, moderate, or strong. This correlation was estimated using SPSS. Any p-value less than 5% was considered statistically significant.

**Ethical considerations and data quality**

The NFHS data is available for public download through the data distribution system of the Demographic and Health Survey (DHS). The study protocol for the NFHS is adapted from the DHS which is reviewed and has been approved by the United States Centers for Disease Control and Prevention. Further, for the NFHS, the ICF International Institutional Review Board and the International Institute for Population Sciences (IIPS) Institutional Review Board reviewed and approved the protocol. All personnel conducting the household survey and administering the tool were trained in standard research protocols including those pertaining to safeguarding vulnerable research subjects. In alignment with the World Health Organization’s (WHO) ethical guidelines (17) on conducting surveys with survivors of domestic violence, only one eligible woman per household was selected for interview on Domestic Violence. Participant information sheet was distributed to each respondent in the appropriate language, and informed consent of all participants was taken during the survey. Dummy questionnaires were used to engage other female/ adult members so that the privacy of the respondent was not compromised. If privacy concerns arose and interview could not be attempted or concluded the interview was not conducted. Additional details on the protocol are available at <https://dhsprogram.com/Methodology/Survey-Types/DHS.cfm>.

**Results**

“Fig 1” presents the heatmaps of each of the three types of IPV as well as combined IPV for all states and UTs. Karnataka, with its highest composite score of IPV, shows up the highest disaggregated burden for each type of IPV. We found that physical IPV is the most common form of IPV for most states. The Western states of India showed up with lighter burdens for all three forms of IPV.

**Fig 1**. State-wise heatmaps of each type of IPV and combined IPV for all states and UTs

In Table 1 we present the composite IPV scores (ranked from highest to lowest) versus the corresponding composite women empowerment score. In the range of composite scores for IPV, the lowest possible was zero, and belonged to Lakshadweep while the highest of three belonged to Karnataka. In other words, Karnataka has the highest burden of IPV while Lakshadweep has the lowest burden. However, when we considered the composite scores of women empowerment alongside each state, we found that empowerment was highest in Puducherry (4.95), closely followed by Telangana and Goa, both with a score of 4.21. Women empowerment scores were lowest for Madhya Pradesh (1.61), closely followed by Bihar (1.77). Women empowerment scores for Karnataka (which has highest IPV burden) was 3.73, and Lakshadweep (which has the lowest IPV burden) was 2.85. (Full table of composite scores for women empowerment and IPV is available as Supporting Information table S2).

**Table 1: Abridged State level Composite Scores for Women Empowerment and IPV**

| **State** | **Intimate Partner Violence**  (from highest to lowest) | **Women  Empowerment** |
| --- | --- | --- |
| Karnataka | 3.0 | 3.73 |
| Bihar | 2.31 | 1.77 |
| Telangana | 1.95 | 4.21 |
| Madhya Pradesh | 1.72 | 1.61 |
| Puducherry | 1.03 | 4.95 |
| Goa | 0.76 | 4.21 |
| Lakshadweep | 0 | 2.85 |

Table 2 shows that overall women empowerment did not show any significant reduction of risk of IPV. However, on the one hand, select components of empowerment, viz. household decision-making (-0.62), own mobile phones (-0.51), and hygienic menstrual practices (-0.45) suggested a strong corresponding reduction in risk of IPV. On the other hand, the component of owning property strongly linked to an increased risk of IPV (0.44). Employment and earning in cash linked to a slight increase in risk of IPV (0.10).

When we disaggregated IPV along its three types (physical, sexual and emotional), we found that the component of household decision-making is associated with a high chance of reduced emotional IPV (-0.70). For employment and earning in cash, risk of sexual IPV was slightly reduced (-0.10). For women owning property the risk of all three types of IPV were increased.

**Table 2: Association of IPV with various independent parameters**

| Spearman Rank Correlation Coefficient | Overall IPV | Physical violence | Sexual violence | Emotional Violence |
| --- | --- | --- | --- | --- |
| Women empowerment | -0.243 (0.153) | -0.086 (0.62) | -0.433 (0.008) | -0.188 (0.272) |
| Decision making | -0.628 (0.000) | -0.402 (0.015) | -0.630 (0.000) | -0.706 (0.000) |
| Employed in last 12 months and earned in cash | 0.101 (0.557) | 0.206 (0.228) | -0.108 (0.530) | 0.179 (0.297) |
| Have own house/land | 0.442 (0.007) | 0.376 (0.024) | 0.328 (0.050) | 0.470 (0.004) |
| Having bank account | -0.065 (0.708) | 0.121 (0.481) | -0.16 (0.350) | -0.154 (0.369) |
| Mobile phone | -0.512 (0.001) | -0.530 (0.001) | -0.507 (0.002) | -0.342 (0.041) |
| Menstrual hygiene | -0.453 (0.005) | -0.261 (0.125) | -0.578 (0.000) | -0.415 (0.012) |

**Discussion**

This study sought to primarily understand the status of women empowerment and its association with IPV against women in India using cross-sectional data from 2019-21. The findings are of the state level, and discussion thus does not focus on women as individuals but as a collective represented at the state level. In this study, women empowerment showed no significant reduction of IPV. However, unpacking women empowerment found that certain components differentially associate with reduced risk of IPV (6).

Increased household decision-making associated with reduced IPV. IPV is a manifestation of abuse of structural power (18); women in power inequitable intimate relationships are very likely to have little to no bargaining or decision-making power (19). Conversely, when women have increased decision-making power, they are likely in a more power equitable relationship. Such relationships are likely to experience reduced IPV. Since the household is the smallest unit of society, household decision-making is “an indicator of power and control within relationships” (11). The present study found that states with higher proportions of women with solo or/and shared household decision-making power also had lowered IPV burden. This is in sync with the above reasoning and also with evidence from other low-and-middle-income countries. A study from Uganda (11) found that women are best protected against IPV when they made household decisions – especially if the decision-making was shared with their husbands. A study from Ethiopia (20) and another from Philippines (21) presented similar arguments, viz. decision-making, when shared, reduces IPV even more than when done only by the woman. The present study, however, did not disaggregate decision-making by solo and shared and so, is unable to comment on this.

Mobile phones associated with reduced IPV. Mobile phones are instruments of social connection and a symbol of empowerment. Women with mobile phones would more likely be connected with safe contacts and a support network. Mobile phones have the symbolic power to contribute to “mitigating women’s fears, sense of isolation, loneliness, and boredom by helping them cope with confinement at home […]” (22). Wide availability of mobiles phones translates to improved financial independence, food security and dietary quality, better household decision-making power by women (22). Pesando further discusses a study that has shown how the use of mobile phones significantly decreased “both men’s and women’s tolerance for IPV and increase[d] women’s autonomy in mobility and economic independence”. Pesando’s study of mobile phones and IPV across 10 LMICs found that women with mobile phones consistently faced reduced IPV risks. The study showed that in households where women had their own mobile phones were likely to be households that had a better recognition of women’s need and rights to share household material resources and have their own networks of friends and families (22).

Good menstrual hygiene strongly associates with reduced IPV – especially with sexual IPV. A study from Kenya (23) found that women who did not have access to safe and hygienic menstrual practices were more vulnerable to IPV. This is likely because communities where menstruation is a taboo, are communities that are rigidly patriarchal and more prone to condoning IPV (24,25). Conversely, women who are aware of hygienic menstrual practices and who use hygienic menstrual products are very likely to have increased exposure to media, access to education, and improved control over their bodies. Such women are likely to enter power-balanced intimate relationships which have reduced chances of IPV.

However, employment increases risk of IPV. One of the predominant theories to explain this is the Male Backlash Theory (9). It explains how society’s normative expectation that women are responsible only for the domestic space stems from centuries of gendered beliefs. Families expect women to be caregivers for children and in-laws and not take up any formal employment (26). Consequently, women who take up employment subvert normative gender roles (9). Further, employed women are more likely to own material and financial resources which also go against traditional gendered expectations and norms (3). These changes disrupt the gender status quo, and women become more likely to face violence (27). For working women accusations of neglecting children and family members, and also of infidelity are known to increase (28). This increases the odds of emotional IPV (29), though some other forms of IPV such as sexual IPV is known to decrease for employed women. This is because working women have reduced contact time with their spouses (who are the IPV abusers) (30). But notwithstanding the increased risk of IPV, employed women are also likely to have their own social support network of peers, friends and work colleagues. These networks become the informal systems that survivors of IPV use for emotional support and help-seeking (31,32). Evidence shows that employed women have higher likelihoods of seeking help for IPV (33).

Property ownership increased risk of IPV. Owning property can be considered similar to employed women building their own material and financial resources. A study from Pakistan (34), found that women owning property had more than twice the risk of IPV, and that husbands’ controlling behaviours intensified when their wives started to own property. Controlling behaviour can manifest as emotional IPV – and in this present study we found that emotional IPV was high for states where proportion of women owing property was high. We also need to understand that the laws of the land, legislations that promote or prevent women’s ownership of property impact the association between property ownership and IPV (35). When legislations support inheritance by women, especially in case of her separation from their husbands, then women’s property ownership is seen to lower risk of IPV. But in societies where property ownership is traditionally a male right, women’s ownership of property is likely to increase IPV (35). The Agricultural Census (2015-16) India estimated that 14 percent of all agricultural land holdings belonged to women. Legislations in India support women’s inheritance but, in several parts of the country, the cultural acceptance of this practice is likely to be still low. It is also possible that while women legally own land, decisions about the land, especially if it an agricultural plot, is taken by the males of the household (36). This effectively implies that land ownership does not translate to women empowerment.

To identify factors that positively associate with reduced IPV, future studies can focus on establishing causality using longitudinal observational studies using national datasets (37). Obiagu (38) reasons that mainstream components of women empowerment such as economic empowerment and education do not directly associate with reduced IPV because “mainstream formal education does not emphasize self-protection against gender-based violence”. This is because normalization of IPV is part of social norms, and as long as the content of education does not train us to question this normalization or teach us the methods of subverting it, only increasing access to education shall not reduce IPV. Obiagu points to evidence that show significant reduction in IPV when intervention programs work with men and boys to change attitudes and perceptions (38).

In this light, future studies could consider systematic reviews of IPV intervention programs that have male engagement as an objective. Also, studies could consider adopting mixed method studies such that big data is tempered with women’s lived experiences of identifying IPV and other contextual factors. Investigating the connection between IPV and environmental factors, such as neighbourhood characteristics and resource accessibility, can offer useful insights (39). Using GIS analysis to understand spatiotemporal dynamics of IPV and its association with programs on women empowerment can help us study how intervention contributes to a reduced burden. Following such a study, we can develop evidence-based interventions that are suited to the unique dynamics of various regions or communities by analysing spatial and temporal patterns.

**Limitations**

The cross-sectional nature of the survey data limits a causal association. The data does not let us assess the extent to which variables are interacting with each other. Further, this study did not look at determinants such as ‘education of husband’ or ‘employment status of husband’ owing to the lack of individual level data at the time of conducting this study.

**Conclusions**

This study sought to understand how components of women empowerment associate with IPV, using data from the most recent round of the NFHS. The study found little reduction in IPV corresponding with improvement in women empowerment. However, the study adds to existing scholarship that demonstrate components of empowerment that associate with decreased burden of IPV. The three top factors associated with reduced IPV are women with household decision-making power, mobile phone ownership and hygienic menstrual practices. The findings of this study would inform future research and intervention that seek to strengthen these specific components of building women empowerment.

[end]

……………………………………………………………………………….

**Acknowledgements**

We sincerely thank the support and solidarity of Shagun Tuli, Rohini Dutta, Minal Shukla, Parth Tailor and Kranti Vora.

**References**

1.	Potter LC, Morris R, Hegarty K, García-Moreno C, Feder G. Categories and health impacts of intimate partner violence in the World Health Organization multi-country study on women’s health and domestic violence. Int J Epidemiol. 2021;50(2):652–62.

2.	Garcia-Moreno C, Jansen HAFM, Ellsberg M, Heise L, Watts C. WHO Multi-Country Study on Women’s Health and Domestic Violence Against Women: Report on the First Results. World Health Organization. 2005;55–89.

3.	Stöckl H, Hassan A, Ranganathan M, M. Hatcher A. Economic empowerment and intimate partner violence: a secondary data analysis of the cross-sectional Demographic Health Surveys in Sub-Saharan Africa. BMC Womens Health. 2021;21(1):1–13.

4.	Bulte E, Lensink R. Empowerment and intimate partner violence: Domestic abuse when household income is uncertain. Rev Dev Econ. 2021;25(1):148–62.

5.	Weitzman A. Women’s and Men’s Relative Status and Intimate Partner Violence in India. Popul Dev Rev. 2014;40(1):55–75.

6.	Schuler SR, Nazneen S. Does Intimate Partner Violence Decline as Women’s Empowerment Becomes Normative? Perspectives of Bangladeshi Women. World Dev [Internet]. 2018;101:284–92. Available from: https://doi.org/10.1016/j.worlddev.2017.09.005

7.	Akter S, Chindarkar N. An Empirical Examination of Sustainability of Women’s Empowerment Using Panel Data from India. Journal of Development Studies [Internet]. 2020;56(5):890–906. Available from: https://doi.org/10.1080/00220388.2019.1605054

8.	Dalal K, Yasmin M, Dahlqvist H, Klein GO. Do electronic and economic empowerment protect women from intimate partner violence (IPV) in India? BMC Womens Health [Internet]. 2022;22(1):1–11. Available from: https://doi.org/10.1186/s12905-022-02110-4

9.	Dhanaraj S, Mahambare V. Male Backlash and Female Guilt: Women’s Employment and Intimate Partner Violence in Urban India. Fem Econ [Internet]. 2022 Jan 2;28(1):170–98. Available from: https://doi.org/10.1080/13545701.2021.1986226

10.	Simister J, Mehta PS. Gender-based violence in India: Long-term trends. J Interpers Violence. 2010;25(9):1594–611.

11.	Zegenhagen S, Ranganathan M, Buller AM. Household decision-making and its association with intimate partner violence: Examining differences in men’s and women’s perceptions in Uganda. SSM Popul Health [Internet]. 2019;8(June):100442. Available from: https://doi.org/10.1016/j.ssmph.2019.100442

12.	Samari G. Education and fertility in Egypt: Mediation by women’s empowerment. SSM Popul Health [Internet]. 2019;9(November 2019):100488. Available from: https://doi.org/10.1016/j.ssmph.2019.100488

13.	Ahuru RR. The influence of women empowerment on maternal and childcare use in Nigeria. Int J Healthc Manag [Internet]. 2021;14(3):690–9. Available from: https://doi.org/10.1080/20479700.2019.1688505

14.	Sell M, Minot N. What factors explain women’s empowerment? Decision-making among small-scale farmers in Uganda. Womens Stud Int Forum [Internet]. 2018;71(October):46–55. Available from: https://doi.org/10.1016/j.wsif.2018.09.005

15.	Iyengar NS, Sudarshan P. A Method of Classifying Regions from Multivariate Data. Econ Polit Wkly [Internet]. 1982;17(51):7–8. Available from: https://www.epw.in/journal/1982/51/special-articles/method-classifying-regions-multivariate-data.html

16.	C Dancey JR. Statistics Without Maths for Psychology. 2017.

17.	Coombs M, Daly K. Putting Women First. Mich Law Rev. 1995;93(6):1686.

18.	Flynn A, Graham K. “Why did it happen?” A review and conceptual framework for research on perpetrators’ and victims’ explanations for intimate partner violence. Aggress Violent Behav [Internet]. 2010;15(3):239–51. Available from: http://dx.doi.org/10.1016/j.avb.2010.01.002

19.	Conroy AA. Gender, Power, and Intimate Partner Violence: A Study on Couples From Rural Malawi. J Interpers Violence. 2014;29(5):866–88.

20.	Ebrahim NB, Atteraya MS. Women’s Household Decision-Making and Intimate Partner Violence in Ethiopia. Academic Journal of Interdisciplinary Studies. 2019;8(2):285–92.

21.	Hindin MJ, Adair LS. Who’s at risk? Factors associated with intimate partner violence in the Philippines. Soc Sci Med. 2002;55(8):1385–99.

22.	Pesando LM. Safer If Connected? Mobile Technology and Intimate Partner Violence. Demography. 2022;

23.	Winter SC, Barchi F. Access to sanitation and violence against women: Evidence from Demographic Health Survey (DHS) data in Kenya. Int J Environ Health Res [Internet]. 2016;26(3):291–305. Available from: http://dx.doi.org/10.1080/09603123.2015.1111309

24.	Cardoso LF, Clark CJ, Rivers K, Ferguson G, Shrestha B, Gupta J. Menstrual restriction prevalence and association with intimate partner violence among Nepali women. BMJ Sex Reprod Health. 2019;45(1):38–43.

25.	Sommer M, Ferron S, Cavill S, House S. Violence, gender and WASH: spurring action on a complex, under-documented and sensitive topic. Environ Urban. 2015;27(1):105–16.

26.	Cerrato J, Cifre E. Gender inequality in household chores and work-family conflict. Front Psychol. 2018;9(AUG):1–11.

27.	Lolayekar AP, Desouza S, Mukhopadhyay P. Crimes Against Women in India: A District-Level Analysis (1991–2011). J Interpers Violence. 2022;37(9–10):NP7289–314.

28.	Stern E, Martins S, Stefanik L, Uwimpuhwe S, Yaker R. Lessons learned from implementing Indashyikirwa in Rwanda- an adaptation of the SASA! approach to prevent and respond to intimate partner violence. Eval Program Plann [Internet]. 2018;71(April):58–67. Available from: https://doi.org/10.1016/j.evalprogplan.2018.08.005

29.	Dalal K. Does economic empowerment protect women from intimate partner violence? J Inj Violence Res. 2011;3(1):35–44.

30.	Zafar S, Saima Zia MS, Amir-ud-Din R. Troubling Trade-offs Between Women’s Work and Intimate Partner Violence: Evidence From 19 Developing Countries. J Interpers Violence. 2021;

31.	Leonardsson M, San Sebastian M. Prevalence and predictors of help-seeking for women exposed to spousal violence in India – a cross-sectional study. BMC Womens Health [Internet]. 2017 Dec 3;17(1):99. Available from: http://bmcwomenshealth.biomedcentral.com/articles/10.1186/s12905-017-0453-4

32.	Malihi Z, Fanslow JL, Hashemi L, Gulliver P, McIntosh T. Factors influencing help-seeking by those who have experienced intimate partner violence: Results from a New Zealand population-based study. Torok M, editor. PLoS One [Internet]. 2021 Dec 23;16(12):e0261059. Available from: http://dx.doi.org/10.1371/journal.pone.0261059

33.	Krishnamoorthy Y, Ganesh K, Vijayakumar K. Physical, emotional and sexual violence faced by spouses in India: evidence on determinants and help-seeking behaviour from a nationally representative survey. J Epidemiol Community Health (1978) [Internet]. 2020 Jun 2;74(9):jech-2019-213266. Available from: https://jech.bmj.com/lookup/doi/10.1136/jech-2019-213266

34.	Murshid NS. An assessment of the association between asset ownership and intimate partner violence in Pakistan. Public Health [Internet]. 2017;150(1994):1–8. Available from: http://dx.doi.org/10.1016/j.puhe.2017.05.004

35.	Peterman A, Pereira A, Bleck J, Palermo TM, Yount KM. Women’s individual asset ownership and experience of intimate partner violence: Evidence from 28 international surveys. Am J Public Health. 2017;107(5):747–55.

36.	Choudhury P. How much land do women own in India? Nobody really knows. Scroll.in [Internet]. 2021; Available from: https://scroll.in/article/989157/how-much-land-do-women-own-in-india-its-still-not-known

37.	Raj A, Silverman JG, Klugman J, Saggurti N, Donta B, Shakya HB. Longitudinal analysis of the impact of economic empowerment on risk for intimate partner violence among married women in rural Maharashtra, India. Soc Sci Med [Internet]. 2018;196(November 2017):197–203. Available from: https://doi.org/10.1016/j.socscimed.2017.11.042

38.	Obiagu AN. Do Women’s Education and Economic Empowerment Reduce Gender-based Violence in Nigeria? [Internet]. 2023. Available from: https://vc.bridgew.edu/jiws/vol25/iss4/12

39.	Stevens HR, Graham PL, Beggs PJ, Hanigan IC. No retreat from the heat: temperature-related risk of violent assault is increased by being inside. International Journal of Urban Sciences. 2023;

**Supporting Information**

**Table S1.
Questions asked to married women on their experience of different forms of IPV:**

| **Type of violence** | **Question asked to married women (18-49 years)** |
| --- | --- |
| **Physical violence** | Did your (last) husband –  – Push you, shake you, or throw something at you? |
|  | – twist your arm or pull your hair? |
|  | – slap you? |
|  | – punch you with his fist or with something that could hurt you? |
|  | – kick you, drag you or beat you up? |
|  | – try to choke you or burn you on purpose? |
|  | – threaten or attack you with a knife, gun, or any other weapon? |
| **Sexual violence** | Did your (last) husband –  – physically force you to have sexual intercourse with him even when you did not want to? |
|  | – physically force you to perform any other sexual acts you did not want to? |
|  | – force you with threats or in any other way to perform sexual acts you did not want to? |
| **Emotional violence** | Did your (last) husband –  – say or do something to humiliate you in front of others? |
|  | – threaten to hurt or harm you or someone close to you? |
|  | – insult you or make you feel bad about yourself? |

**Table S2.
Composite IPV scores (ranked from highest to lowest) versus the corresponding composite women empowerment score:**

| **State** | **Intimate Partner Violence**  (from highest to lowest) | **Women  Empowerment** |
| --- | --- | --- |
| Karnataka | 3.0 | 3.73 |
| Bihar | 2.31 | 1.77 |
| West Bengal | 2.04 | 2.11 |
| Telangana | 1.95 | 4.21 |
| Uttar Pradesh | 1.87 | 2.05 |
| Assam | 1.84 | 2.39 |
| Ladakh | 1.82 | 3.65 |
| Manipur | 1.74 | 4.03 |
| Jharkhand | 1.73 | 2.78 |
| Madhya Pradesh | 1.72 | 1.61 |
| NCT of Delhi | 1.62 | 3.07 |
| Arunachal Pradesh | 1.60 | 3.72 |
| Maharashtra | 1.56 | 2.61 |
| Andhra Pradesh | 1.55 | 3.15 |
| Odisha | 1.51 | 3.01 |
| Tamil Nadu | 1.46 | 4.75 |
| Tripura | 1.43 | 1.93 |
| Meghalaya | 1.38 | 3.28 |
| Rajasthan | 1.36 | 2.17 |
| Haryana | 1.12 | 2.45 |
| Chhattisgarh | 1.08 | 2.87 |
| Sikkim | 1.05 | 3.87 |
| Puducherry | 1.03 | 4.95 |
| Uttarakhand | 0.91 | 2.83 |
| DDN and Daman & Diu | 0.89 | 3.89 |
| Gujarat | 0.83 | 2.28 |
| Jammu & Kashmir | 0.80 | 2.81 |
| Goa | 0.76 | 4.21 |
| Punjab | 0.73 | 3.67 |
| Himachal Pradesh | 0.56 | 3.38 |
| Mizoram | 0.55 | 3.80 |
| A & N Islands | 0.54 | 3.83 |
| Kerala | 0.54 | 3.63 |
| Chandigarh | 0.52 | 3.60 |
| Nagaland | 0.43 | 2.93 |
| Lakshadweep | 0 | 2.85 |

**[end of document]**

1. This is a major change from previous NFHS rounds. Till the fourth round of the survey (2015-16), domestic and spousal violence/IPV questions were asked to married women in the age group 15-49 years. From the current fifth round, the age group has been changed to 18-49 years. This was done to avoid the legal mandate that bounds any person with the knowledge of child sexual abuse to report it to the authorities (as per the Protection of Children from Sexual Offences, 2012). Since under the changed law, a woman under the age of 18 years is legally a minor, any information about her sexual life, even if with her husband would require reporting to the authorities. However, India does not recognise marital rape as a legal offence; so, any reporting of sexual abuse by the husband (of a wife who is younger than 18 years) would amount to arrest of the man and sentencing as per the rape laws in the country.
